# Defocus Discrimination in Video: Motion in Depth

**DOI:** 10.1177/2041669517737560

**Published:** 2017-11-21

**Authors:** Vincent A. Petrella, Simon Labute, Michael S. Langer, Paul G. Kry

**Affiliations:** School of Computer Science, 5620 McGill University, Quebec, Canada

**Keywords:** blur, perception, defocus, depth of field, motion in depth

## Abstract

We perform two psychophysics experiments to investigate a viewer’s ability to detect defocus in video; in particular, the defocus that arises in video during motion in depth when the camera does not maintain sharp focus throughout the motion. The first experiment demonstrates that blur sensitivity during viewing is affected by the speed at which the target moves towards the camera. The second experiment measures a viewer’s ability to notice momentary defocus and shows that the threshold of blur detection in arc minutes decreases significantly as the duration of the blur increases. Our results suggest that it is important to have good control of focus while recording video and that momentary defocus should be kept as short as possible so it goes unnoticed.

## Introduction

In photography, depth of field (DOF) refers to the range of depths in a scene where objects appear in focus. A common photographic technique is to manipulate the DOF to bring more attention to some objects than others. Such effect is likewise used in cinematography, where it is also common to track an object as it moves in depth. The act of changing the plane of focus over time is known as *focus pulling*. One of the most challenging jobs on a movie set is that of the first camera assistant who pulls the focus on certain actors or objects throughout a shot. Positions and distances can be established in rehearsal, but focus pulling remains difficult because of natural random variation in timing and motion. Shallow DOF makes this task even more challenging, namely when using a wide open aperture to produce dramatic blurry backgrounds. The DOF can be in the order of centimeters when using medium length or telephoto lenses, but also when filming at close distances with a wide angle lens. In these cases, focus pulling must be done with great care, as a small error can be the difference between focusing on the actor’s ears instead of their eyes.

In general, two types of blur can occur when one views a video. One is the *defocus blur* that arises from errors in focus pulling. It can happen if the focus is constant and the object moves in depth or if one makes an error when pulling focus on an object moving in depth. In either case, such defocus blur can be present either throughout a shot or momentarily, as the object’s depth and focal depth vary. The second type of blur is the *motion blur* that occurs either in the video, when the image of an object moves across the sensors, or in the visual system when the image of an object moves across the retina. This motion blur is due to a finite integration time of the photoreceptors, either in the camera capturing the video or in the eye observing the displayed video, respectively.

Most studies of blur discrimination have been for static stimuli only, that is, defocus blur. A well-known finding is that blur discrimination thresholds at large reference blurs obey roughly a Weber law, so just noticeable differences (JNDs) in blur are proportional to a reference blur level. At small blur references, blur discrimination thresholds exhibit a dipper function. This dipper function exists both in the fovea and in the periphery (Maiello, Walker, Bex, & Vera-Diaz, 2017; Wang & Ciuffreda, 2005). In the fovea, for example, the dip typically occurs near 1 arcmin of blur. For an excellent review of this topic for the case of static stimuli, see Watson and Ahumada (2011).

Previous studies on blur perception of moving objects address lateral motion only. These studies concentrate on the curious perceptual phenomenon that moving patterns appear sharper than they should, given the motion blur in the visual system. It has been argued (Burr & Morgan, 1997) that this illusory sharpness may be due to the elevation of blur discrimination thresholds for moving patterns (Pääkkönen & Morgan, 1994), rather than to a motion sharpening process within the visual system, as it has been proposed by other authors. Regardless of the underlying mechanism, it is important to keep in mind why the task of blur discrimination for lateral motion is inherently difficult, namely it requires disentangling any defocus blur in the pattern from the motion blur that occurs within the visual system.

In this article, we address the related, but different question of when, if at all, does a viewer notice defocus in a video of an object that moves in depth. The experiments that we present below are to our knowledge the first to consider this question. We hypothesize that viewers are less sensitive to defocus of objects that are approaching (expanding) than to objects that are static. We carry out two experiments to explore this hypothesis. The first measures how well an observer can discriminate a constant level of blur in a uniformly expanding pattern. Our results show that faster expansion rates yield higher blur discrimination thresholds. Our second experiment considers the case of an object that is moving in depth and that may momentarily be out of focus, for instance, when the object moves unpredictably as in the case of focus pulling. Our results show that defocus is more difficult to detect when it occurs over shorter durations. In both experiments, we assume that there is no motion blur within each frame of the video by enforcing an infinitesimal exposure time for each frame, akin to an instantaneous shutter speed. Motion blur, however, may still be present in the visual system.

## Experiment 1: Defocus Discrimination for Constant Expansion

Our first experiment measures how well observers can discriminate a constant level of blur in a uniformly expanding pattern.

### Method

#### Observers

Seven naive subjects participated in the experiment. All had normal or corrected-to-normal visual acuity.

#### Apparatus and stimuli

**Figure 1.**
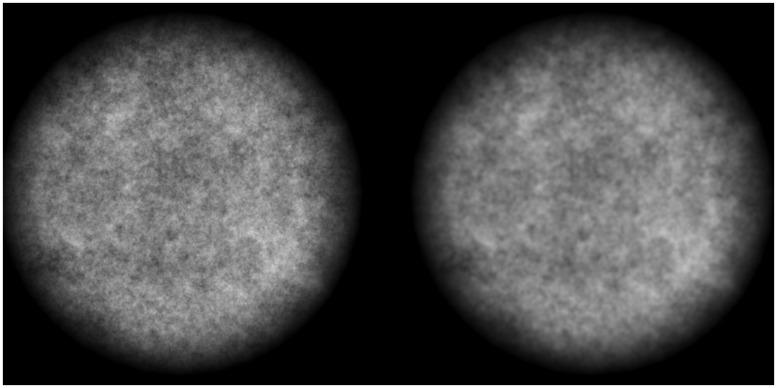
Screen shot mid-trial of the first experiment. The participant is tasked to determine which side is blurrier. The images scale at a constant rate.

Each trial consisted of a pair of image sequences which underwent a two-dimensional scaling expansion at a constant rate. An example stimulus frame (still image) is shown in Figure 1. In both the left and right halves of the frame, the texture was a fractal 1/f noise pattern (similar appearance at all scales). We used a single precomputed periodic texture (4,096 pixels square) with a trilinearly sampled MIP map. The texture was randomly rotated and translated for each trial to reduce familiarity effects. The left and right halves were windowed to smoothly blend to a constant background color at the boundary. The left and right sequences in each trial were identical except that one contained more blur than the other. The subject’s task was to choose which had more blur. The defocus was rendered with a Gaussian kernel. While it does not perfectly simulate optical blur, as would a realistic lens and aperture model, it is separable and fast to compute, allowing for real-time renderings of stimuli at a resolution of 1920-by-1080 pixels at up to 144 frames per second (fps). We employed a two-pass shader using a 51 pixel wide kernel. For each blur level, we used a normalized discretized Gaussian that matched the desired standard deviation.

The blur in the left and right half of each frame was rendered separately. One randomly chosen side was blurred at the reference (pedestal) blur level and the other side (the test) had a higher blur level. Letting $\sigma_{\text{ref}}$ and $\sigma_{\text{test}}$ be the standard deviations of the Gaussian blur for the reference and test, we define the blur difference as

$$\Delta \sigma \equiv \sigma_{\text{test}}-\sigma_{\text{ref}}$$

The four reference blur levels were 0.5, 1.6, 3.2, and 4.8 arcmin. The choice of Δσ on each trial will be explained below.

Observers were seated at a distance of 150 cm from a high-definition 24 in. monitor (HP ZR24w) refreshing at 60 fps. The stimulus on the display was 32.6 cm wide or about 1.6 pixels per arcmin. The left and right stimuli were each just under 5° of visual angle. This viewing angle and resolution defines the *standard viewing* scenario for this article.

We used four scaling per millisecond rates: 1 (static, i.e., no expansion), 1.001 (slow), 1.002 (fast), and randomly ordered frames (flicker). Making a small angle approximation, a point at *θ* degrees of visual angle from the center of expansion goes to 1.001θ degrees in 1 ms for slow expansion or 1.002θ for fast expansion. The corresponding image speed at *θ* degrees is *θ* deg/s or 2θ deg/s, respectively. This is in the range of speeds used by Pääkkönen and Morgan (1994) in their study of the effects of motion on blur discrimination. Flicker is the case of very fast expansion combined with very fast shutter speed, such that the camera would not capture a fast-moving object at a high enough sampling rate and the video would just appear as uncorrelated sequences of images.

#### Procedure

There were 16 stimulus conditions, namely four reference blurs and four motions. Each participant was shown 30 stimuli for each condition, for 480 trials in total. Conditions were randomly interleaved.

In addition to the 480 trials for each subject, catch trials were added, which consisted of a stimulus of zero reference blur on one side and a high blur on the other. In a pilot study, we also tested contracting patterns that simulate motion away from the camera. The results appeared to be similar. Thus, to make the best of a limited number of trials per subject, we eliminated contracting patterns in the experiment. For each condition, the blur levels from trial to trial were determined by a 1-up/2-down adaptive staircase method (Kingdom & Prins, 2009). The increments and decrements were chosen such that the blur levels tended to be distributed near those for which the observer is 75% correct.

At the start of each trial, the word “ready” was shown for 800 ms followed by the image sequences for 2 seconds followed by a black screen until the observer responded. Subjects were free to make eye movements during each trial.

### Results

**Figure 2.**
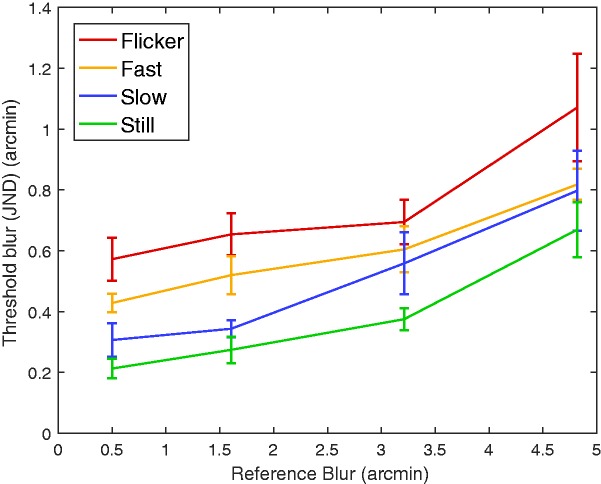
Results from Experiment 1. Mean blur discrimination thresholds (JNDs) and the standard error of the mean over the subjects are plotted for each reference blur. Mean thresholds increase with reference blur. Thresholds also are higher for faster speeds and for randomized frames (flicker). Overall, blur discrimination performance is very good.

For each observer and each condition, we estimated the threshold (JND) by the average levels of the blur increment at the last six reversals of the staircase. We ensured that all conditions had reversed at least six times to include the results for the participant. Figure 2 illustrates an increase in JND thresholds as scaling rates increase. We analyzed the thresholds using a two-way repeated measures analysis of variance (ANOVA), for which the results can be found in Table 1. The mean of the motion conditions was significantly different, F(3,18)=13.715,p<0.0005. This was expected since motion produces retinal blur that is known to raise thresholds (Burr & Morgan, 1997; Pääkkönen & Morgan, 1994). For the flicker effect, subjects could not track points from frame to frame and thus were not able to compare areas between the left and right stimuli. This could explain the higher thresholds for this condition. JNDs also rose with the reference blur, F(1.216,7.296)=33.954,p<.0005 using Greenhouse-Geisser correction. Again, this was expected given previous results with static stimuli (Watson & Ahumada, 2011). We did not observe a dipper function, presumably since we did not consider the case of zero reference blur. For the range of reference blurs that we examined, blur thresholds increased as the reference blur and stimulus velocities increased. An important difference is that they study lateral motion with fixed gaze, whereas in our experiment eye movements were not restricted. Our participants were thus allowed to look at the center of expansion, which does not exhibit motion. We found that the expansion rate was a significant factor in our results. This suggests that either participants did not choose to gaze only at this center of expansion or that they did use the center of expansion but the nonmoving region was smaller for the faster stimulus, providing less information. Finally, we observe no statistically significant interactions between the different conditions tested in this experiment.

**Table 1.** Results of the Two-Way Repeated Measures ANOVA From Experiment 1 Between the Expansion Rate and Reference Blur Conditions.

Factors	Type III SS	*df*	Mean square	*F*	*p*
**Expansion Rate**	1.991	3	0.664	13.715	**.000**
Error(Expansion rate)	0.871	18	0.048	N/A	N/A
**Reference Blur**	3.437	1.216	2.826	33.954	**.000**
Error(Reference blur)	0.607	7.296	0.083	N/A	N/A
Expansion Rate × Reference Blur	0.111	2.531	0.044	0.416	.713
Error(Expansion Rate × Reference Blur)	1.603	15.19	0.106	N/A	N/A

*Note.* The significant effects are highlighted in boldface. ANOVA = analysis of variance.

In synthetic videos that do not have any motion blur within each frame, akin to filming with instantaneous camera shutters, subjects are less sensitive to blur for expanding stimuli as the expansion speed increases. These results are consistent with previous studies of blur discrimination in video, which only considered lateral motion.

## Experiment 2: Defocus Detection During Abrupt Motion Change

This second experiment investigates gradual defocus introduced momentarily in video. We examine how well observers can detect these effects that potentially coincide with a change in the motion in depth of the stimulus. This detection experiment examines how well subjects can discriminate a stimulus in which defocus blur is present in the video from one with no defocus blur present in the video.

### Method

In a pilot study, we found that step changes in defocus were detected easily, whether from sharp to blurry or vice versa, for both static and expanding stimuli. Here, we investigate blurring over short time durations in various motion and texture conditions.

#### Observers

Six naive observers participated in this study. All had normal or corrected-to-normal visual acuity.

#### Apparatus and stimuli

The stimuli came in two forms: no motion and expansion then stop. We defined image blurring as being gradual, increasing magnitude over multiple milliseconds then decreasing symmetrically to 0.

For stationary stimuli, blur could happen at a random time during the trial. For expanding stimulus, blur occurred at a random time coinciding with the moment when the stimulus stopped expanding. To cover general expansion conditions, stimuli expanded similarly to our first experiment, with rates chosen randomly between 1.4θ deg/s and 2θ deg/s for each trial.

**Figure 3.**
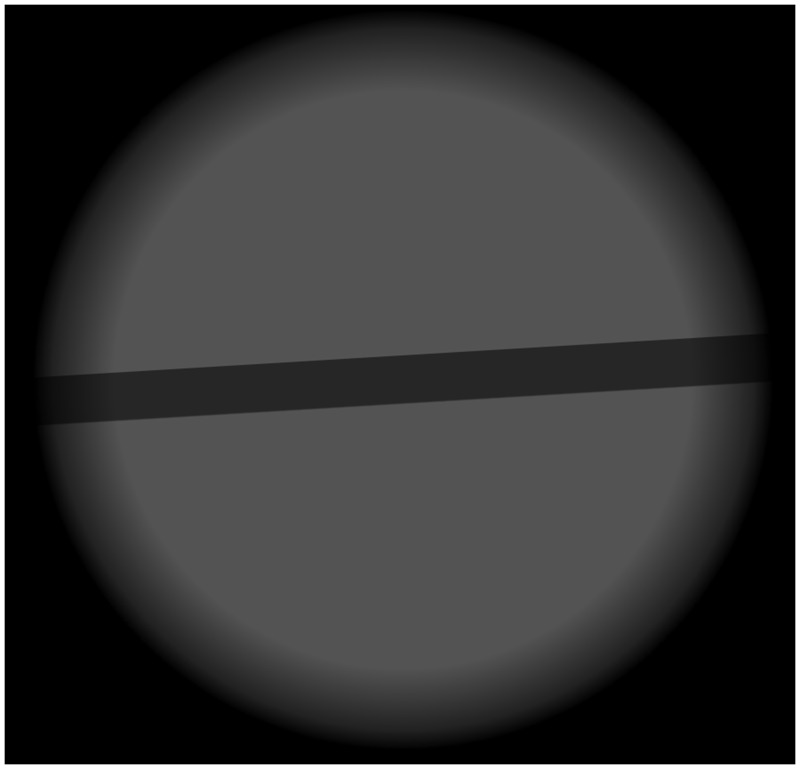
Screen shot mid-trial of the second experiment, Showing an expanding straight bar.

Two texture conditions were used and randomly interleaved: The 1/f noise condition from Experiment 1 and a second condition consisting of a straight bar centered in the middle of the texture, randomly tilted to prevent the participant from becoming accustomed to gazing at a particular area while viewing the stimulus. An example is shown in Figure 3. In the blurred stimuli, the blur magnitude followed a temporal hat function during the randomly inserted blur interval and had otherwise 0 arcmin of blur over the entire 1.5 seconds (i.e., from the start time of the blur interval, it increased from 0 arcmin to the test peak blur in the first half of the inserted interval and then decreased back to 0). Six blur durations from approximately 7 ms to 444 ms were tested. These corresponded to 1, 2, 4, 8, 16, and 64 frames at 144 fps. To sample the hat function for the 1 and 2 frames of blur cases, we sampled the peak blur magnitude once and twice, respectively.

For this experiment, we presented the stimuli on a monitor refreshing at 144 fps (BENQ XL2411). This enables us to display momentary blur for very short durations. Other than changing the monitor, the viewing conditions remained the same as in the first experiment.

#### Procedure

In each trial, subjects were shown two similar stimuli one after the other, with the only difference being that one, chosen randomly at each trial, was blurred momentarily and the other was not. Once both stimuli were displayed, the subjects had to identify which stimulus had been momentarily blurred. In the case where they were unable to notice blur in either stimulus, the subjects were instructed to choose randomly. Each trial consisted of a reference and a momentarily blurred stimulus for 1500 ms each, separated by a blank interval of 100 ms.

We used a weighted 1-up/2-down method to estimate the detection threshold values. We ran a pilot study to determine an approximate value for these detection thresholds, which we used to initialize our staircases for faster convergence. For each type of stimulus, we waited to observe 14 reversals on the staircase before termination. Thresholds in each condition were then computed by taking the mean blur levels for the last 12 reversals in that condition. Due to the peculiar nature of the task, participants were first presented with a short tutorial displaying exaggerated blur values to illustrate the kinds of visual artifacts they could expect to see.

### Results

**Figure 4.**
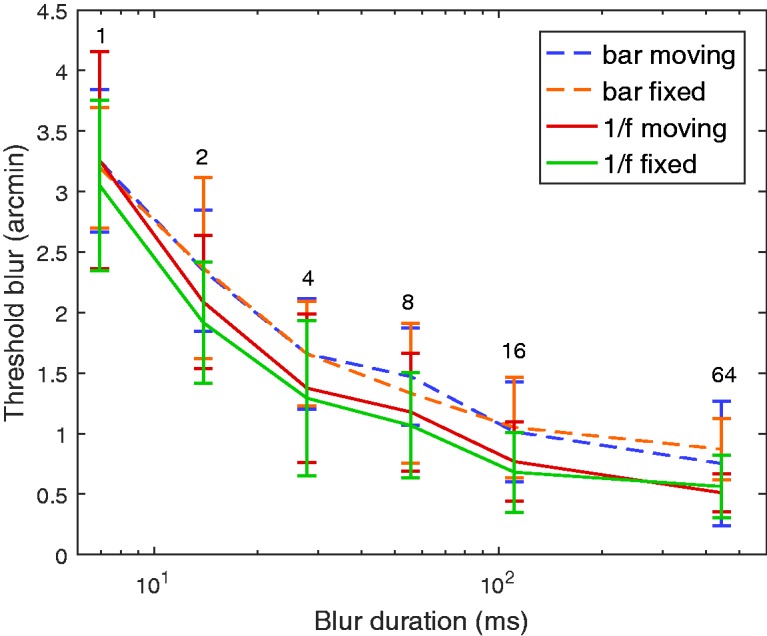
Results from Experiment 2 showing that blur detection thresholds fall as blur durations increase. Mean (and standard error of the mean) thresholds over the subjects are plotted with time on a *log* scale for clarity. The corresponding blur duration as a number of frames at 144 fps is displayed above the curves.

The results are shown in Figure 4. As the blur duration is increased, the sampled blur becomes easier to detect. We employed three-way repeated measures ANOVA (see Table 2). We cannot conclude that the means between motion conditions are different, F(1,5)=2.593,p=.168. We can, however, conclude a difference in the means between the duration conditions, F(5,25)=192.539,p<.0005. Subjects needed a larger amount of blur over shorter time durations to detect the momentary blur. Furthermore, the 1/f noise texture yields more noticeable momentary blur than the horizontal bar, F(1,5)=61.643,p=.001. We hypothesize that the spatially sparse information in this stimulus (blur cues being localized on the edges of the bar) reduced the number of image points containing blur. Finally, we find no significant interaction between the three effects tested in this second experiment.

**Table 2.** Results of the Three-Way Repeated Measure ANOVA From Experiment 2 on the Texture, Motion, and Duration Conditions.

Factor	Type III SS	*df*	Mean square	*F*	*p*
Motion	0.253	1	0.253	2.593	.168
Error(motion)	0.487	5	0.097	N/A	N/A
**Duration**	268.121	5	53.624	182.539	**.000**
Error(Duration)	7.344	25	0.294	N/A	N/A
**Texture**	6.666	1	6.666	61.643	**.001**
Error(Texture)	0.541	5	0.108	N/A	N/A
Texture × Motion	0.220	1	0.220	21.690	.006
Error(Texture × Motion)	0.051	5	0.010	N/A	N/A
Texture × Duration	0.793	5	0.159	1.247	.317
Error(Texture × Duration)	3.181	25	0.127	N/A	N/A
Motion × Duration	0.485	5	0.097	1.442	.244
Error(Motion × Duration)	1.683	25	0.067	N/A	N/A
Texture × Motion × Duration	0.110	5	0.022	0.373	.862
Error(Texture × Motion × Duration)	1.471	25	0.059	N/A	N/A

*Note.* The significant effects are highlighted in boldface. ANOVA = analysis of variance.

One minor point to note is that the threshold values in Figure 4 should not be directly compared with those of Figure 2 because of the differences in experimental setup and tasks involved (detection versus discrimination).

## Discussion

Our results could potentially be used in applications requiring an understanding of sensitivity to defocus occurring when objects move out of focus. Here, we discuss our experimental results in the context of different applications and speculate on broader implications related to new technologies and other research.

### Application to Auto Focus Systems

There exist various methods to automate focus pulling, but they do come with shortcomings. For example, most consumer photography cameras use phase detection to auto focus. While suited for pulling focus on static objects, typical implementations fail to deliver fast and reliable enough focus on moving objects. There is, however, another practical solution for difficult focus pulling scenarios. Using a motion capture system to measure the location of actors and objects, one can drive the camera focus automatically. The Andra Radius follow focus system, recently available from Cinema Control Labs is one such implementation. While this approach may trivially produce sharp focus of the target when everything is static, the end to end delay from measurement to control of the focal plane will result in a soft defocus of the target whenever the camera or target moves in a way that produces motion in depth. This latency exists in all motion acquisition systems. For instance, magnetic tracking systems, while ideal for this application because the sensors can be hidden on actors and objects, typically contribute to at least 15 ms of latency (Jones, 2012). Filtering, communication, and motor control are all additional sources of delay. In our first experiment, we showed that people are sensitive to constant defocus in video. Thus, our findings suggest that it is important to improve tracking by compensating for motion capture latency, as the defocus that this delay produces when the system focuses on an outdated position in depth will likely be noticeable.

While it is possible to filter out this delay with knowledge of the object’s motion, there is no current solution that will consistently produce accurate enough prediction to avoid substantial focusing errors in the final video. Such defocus is most exacerbated during abrupt motion changes. In our second experiment, we showed that the kinds of momentary focusing errors that may arise from these situations may likely be perceptible to the human eye. We report measurements of thresholds of blur detection as a function of the duration of the momentary blur. These results could provide a benchmark to test the quality of techniques that may be developed to improve on these types of defocus errors.

Light-field photography is a solution that avoids the problem entirely. Light-fields capture incident light that can later be refocused in a postprocessing step (Ng, 2005). There are light-field video cameras targeted at industrial applications, with video capabilities (such as the Raytrix R8), though the resolution and image quality of such cameras are insufficient for most entertainment applications. The Lytro Cinema system, in contrast, is able to shoot high-definition light-field videos, but the complexity and cost of the system are probably impractical for most cinematography applications.

### Applications to Augmented and Virtual Reality Systems

In the emerging research on augmented and virtual reality, defocus has also been considered to enhance viewing comfort and realism. Vinnikov and Allison (2014) investigate the use of gaze-contingent depth-of-field simulation, in which a real-time render is blurred according to where the user is looking in the image, to resemble the blur from accommodation. As in the work of Mauderer, Conte, Nacenta, and Vishwanath (2014), the authors claim that the effect enhanced perception of depth on common displays, but it did not help in the presence of stereoscopic cues (using three-dimensional displays). Furthermore, they found that a subjective measure of viewing comfort was impaired by the effect, which seems to contradict the reports of O’Hare, Zhang, Nefs, and Hibbard (2013). Duchowski et al. (2014) find that the technique does, however, reduce visual discomfort in stereoscopic viewing, while still being reportedly disliked by participants in the studies. Finally, Maiello, Chessa, Solari, and Bex (2014) use gaze-contingent depth of field with optical blur added to light-field photographs viewed on a stereoscopic display. Their work suggests that the addition of the blurring effect helped with achieving binocular fusion, most dramatically with participants who originally struggled at this task.

Similarly to motion capture, gaze tracking systems currently induce significant latency (Saunders & Woods, 2014). Momentarily defocus, therefore, arises when the viewer’s eyes settle on an object of interest as the system estimates the gaze and renders blur. The results from our second experiment may provide insight on the impact of such delay with blur renders of different magnitudes and may hint at a benchmark for lag compensation methods.

### Depth Perception and Defocus Blur

Defocus blur has long been used for enhancing perceived depth in photography and in computer graphics, although surprisingly few perceptual studies have been done. It has been shown, for example, that blur gradients provide perceptual cues about scene scale and may explain the tilt-shift illusion effects (Held, Cooper, O’Brien, & Banks, 2010; Vishwanath & Blaser, 2010). There is some evidence that blur can help determine depth order at occlusion boundaries (Mather, 1997; Mather & Smith, 2002), although the effect size is relatively weak for rendered blur in comparison with optical blur (Zannoli, Love, Narain, & Banks, 2016). Blur also can be combined with other depth cues. Mather (1997) hypothesized that defocus blur cues might be complementary to binocular disparity, namely the visual system may use disparity cues near fixation and blur cues away from fixation. Held, Cooper, and Banks (2012) found evidence to support this hypothesis using a volumetric stereoscopic display, although Vishwanath (2012) challenged the interpretation of these experiments, claiming that Held et al. (2012) measured blur discrimination thresholds rather than perceived depth from blur. Langer and Siciliano (2015) used a traditional stereo display with simulated blur but were not able to reproduce the results of Held et al. (2012). Maiello, Chessa, Solari, and Bex (2015) further investigated the issue using light-field photographs to blur pictures in postprocessing. They found that depth discrimination performance was highest in the presence of geometric and disparity cues but blur cues impaired performance.

One open and interesting question that is raised by our experiments is whether the visual system combines blur cues with motion cues to depth. For example, motion parallax that is due to lateral observer motion provides similar depth information to binocular disparity, and it is well known that the visual system combines these cues. We might not expect motion parallax to be complementary to blur in the same way that binocular disparity may be complementary to blur, since there is no analogous binocular fusion problem with large motion parallax. However, there may be other interesting effects that occur when blur and motion parallax are combined, such as at occlusion boundary. A question that is more directly related to our experiments is whether there is an interaction between time varying blur and motion in depth. For example, does an expanding pattern tend to appear more or less as a motion in depth if it undergoes a blur change that is consistent or inconsistent with motion in depth?

## Conclusion

We present two psychophysics experiments to investigate a viewer’s ability to detect defocus in video. To our knowledge, no previous studies have investigated blur discrimination when viewing an object moving in depth. The result of the first experiment shows how well observers can discriminate constant defocus when viewing a video of an object moving in depth, specifically an expanding image pattern. We show that faster expansion speed reduces sensitivity to blur. These results prove consistent with previous work on blur discrimination for lateral motion in video. In our second experiment, we demonstrate that observers require larger amounts of blur to detect a shorter duration increase and decrease in defocus blur. By using a high refresh rate monitor, we are able to measure these thresholds for a wide range of defocus durations. We also discuss the potential application of our results to new cinematography methods and graphics applications, namely providing benchmarks of focus quality for films and augmented reality systems. We finally relate our work to previous studies of blur for depth and motion perception.
